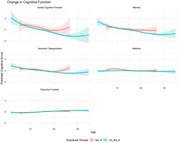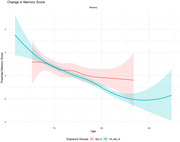# Cognitive function following dipeptidyl peptidase 4 inhibitors use in older adults with type 2 diabetes: A nationwide cohort study

**DOI:** 10.1002/alz70860_106328

**Published:** 2025-12-23

**Authors:** Avi Cohen, Stephen Z Levine, Shiraz Vered, Galit Weinstein

**Affiliations:** ^1^ School of Public Health, University of Haifa, Haifa, Mount Carmel, Israel

## Abstract

**Background:**

Dipeptidyl peptidase 4 inhibitors (DPP‐4i) are widely used antidiabetic medications with reported neuroprotective potential beyond their glycemic control effects. Nonetheless, the association between DPP‐4i use and cognitive function is unclear. We examined the association between the use of DPP‐4i and change in cognitive function in older adults with type 2 diabetes (T2D).

**Method:**

This study included, at cohort entry, non‐demented T2D older adults (≥65y), from the Israel Diabetes and Cognitive Decline (IDCD) study, who had repeated cognitive assessments of memory, attention, executive functions, and semantic categorization. Detailed information on antidiabetic medication use periods was ascertained from electronic health records from a Health Maintenance Organization. We classified exposure groups based on DPP‐4i and combination use at least one year before the first cognitive assessment versus non‐use. We fitted a longitudinal linear mixed‐effects model with an interaction term between medication group and age to quantify the association between antidiabetic medication groups and cognitive change. Models were adjusted for multiple potential confounders, including changes in body mass index, Haemoglobin A1C (HbA1C), lipids, cardiovascular disease diagnosis, and diabetes duration.

**Result:**

Overall, 775 participants were included (mean age 72.4±4.7y; 60% women). Of the total sample, 90 participants (11.6%) were in the DPP‐4i group at cohort entry. The mean T2D diagnosis age was 61.8±5.2y in the DPP‐4i group vs. 64.8±5.6y in the non‐DPP‐4i group. The follow‐up times were similar in both groups (DPP‐4i: 4.4±1.8y vs. non‐DPP‐4i: 4.7±1.9y). Additionally, mean HbA1c levels (%) at cohort entry were 7.3±1.3 in the DPP‐4i group vs. 6.8±0.9 in the non‐DPP‐4i group, respectively. No differences were observed in global cognition, attention, executive function, and semantic categorization. A potential trend of slower memory decline was observed in the DPP‐4i group compared to the non‐DPP‐4i group, with the effect varying by age (β=0.11±0.06; *p* = 0.06). Notably, over the age of 70 memory score curves began to diverge.

**Conclusion:**

In this nationwide cohort study, DPP‐4i was not associated with change in cognitive functions, but a trend in memory was observed. These results suggest that additional research with a larger sample and a longer follow‐up time is required to further explore these results.